# Imaging of alert patients after non-self-inflicted strangulation: MRI is superior to CT

**DOI:** 10.1007/s00330-023-10354-3

**Published:** 2023-11-13

**Authors:** Thomas D Ruder, Alexandra Gonzenbach, Jakob Heimer, Leonie Arneberg, Jolanta Klukowska-Rötzler, Simone Blunier, Aristomenis K Exadaktylos, Wolf-Dieter Zech, Franca Wagner

**Affiliations:** 1 Institute of Diagnostic, Interventional and Pediatric Radiology, InselspitaI, Bern University Hospital, University of Bern, CH-3010 Bern Freiburgstrasse, Switzerland; 2 Department of Emergency Medicine, InselspitaI, Bern University Hospital and University of Bern, Bern, Switzerland; 3 Department of General Surgery, Hospital Linth, Uznach, Switzerland; 4 Department of Mathematics, Seminar for Statistics, ETH Zurich, Zurich, Switzerland; 5 Department of Medicine, Hospital Emmental, Burgdorf, Switzerland; 6 Institute of Forensic Medicine, University of Bern, Bern, Switzerland; 7 Institute of Diagnostic and Interventional Neuroradiology, Inselspital, Bern University Hospital, University of Bern, Bern, Switzerland

**Keywords:** Emergency radiology, Non-fatal strangulation, Non-accidental injury, Larynx fracture, Blunt cerebrovascular injury

## Abstract

**Objective:**

To assess the accuracy of CT and MRI reports of alert patients presenting after non-self-inflicted strangulation (NSIS) and evaluate the appropriateness of these imaging modalities in NSIS.

**Material and methods:**

The study was a retrospective analysis of patient characteristics and strangulation details, with a comparison of original radiology reports (ORR) to expert read-outs (EXR) of CT and MRI studies of all NSIS cases seen from 2008 to 2020 at a single centre.

**Results:**

The study included 116 patients (71% women, *p *< .001, *χ*^2^), with an average age of 33.8 years, mostly presenting after manual strangulation (97%). Most had experienced intimate partner violence (74% of women, *p *< .001, *χ*^2^) or assault by unknown offender (88% of men, *p *< 0.002 *χ*^2^). Overall, 132 imaging studies (67 CT, 51% and 65 MRI, 49%) were reviewed. Potentially dangerous injuries were present in 7%, minor injuries in 22%, and no injuries in 71% of patients. Sensitivity and specificity of ORR were 78% and 97% for MRI and 30% and 98% for CT. Discrepancies between ORR and EXR occurred in 18% of all patients, or 62% of injured patients, with a substantial number of unreported injuries on CT.

**Conclusions:**

The results indicate that MRI is more appropriate than CT for alert patients presenting after non-self-inflicted strangulation and underline the need for radiologists with specialist knowledge to report these cases in order to add value to both patient care and potential future medico-legal investigations.

**Clinical relevance statement:**

MRI should be preferred over CT for the investigation of strangulation related injuries in alert patients because MRI has a higher accuracy than CT and does not expose this usually young patient population to ionizing radiation.

**Key Points:**

*• Patients presenting after strangulation are often young women with a history of intimate partner violence while men typically present after assault by an unknown offender.*

*• Expert read-outs of CT and MRI revealed potentially dangerous injuries in one of 14 patients.*

*• MRI has a significantly higher sensitivity than CT and appears to be more appropriate for the diagnostic workup of alert patients after strangulation.*

## Introduction

In recent years, the number of publications on non-fatal strangulations has grown substantially [1–6]. This growth may be the result of a greater general awareness of domestic violence and of the role of radiology in non-accidental injuries in adults [6–9]. There is agreement in the literature that most patients presenting with strangulation-related injuries after assaults are young women [1–5, 10], and that most survivors of strangulation have no or only minor injuries [1–6, 11]. Potentially dangerous injuries, such as fractures of the hyoid-larynx complex (HLC) and blunt cerebral vascular injuries (BCVI), which may lead to compromised airways and hypoxic brain injury, respectively, are rare in people who have survived strangulation [1–6, 10, 12]. The pooled prevalences of HLC fractures and BCVI on imaging in eight previous studies on NFS are 0.4% and 1.3%, respectively (Table 1) [1–6, 10, 12].

**Table 1:** Prevalence of reported HLC fractures and BCVI after survived strangulation

1st author	Year	All cases	Comparable cases^a^	HLC^b^ fracture		BCVI^c^	
Yen [12]	2007	14	14	0	0.0%	0	0.0%
Christe [10]	2009	56	56	0	0.0%	0	0.0%
Heimer [1]	2019	114	114	1	0.9%	0	0.0%
Zuberi [2]	2019	142	133	0	0.0%	3	2.3%
Matusz [3]	2019	349	187	0	0.0%	2	1.1%
Williamsson [4]	2021	425	101	0	0.0%	2	2.0%
MacDonald [5]	2021	209	13	0	0.0%	2	15.4%
Bergin [6]	2022	345	216	2	0.9%	2	0.9%
Total		1654	834	3	0.4%	11	1.3%

^a^Comparable cases = cases comparable to the population of this study, i.e. patients in whom imaging of the cervical vessels was performed after manual or ligature strangulation. ^b^*HLC*, hyoid-larynx-complex; ^c^*BCVI*, blunt cerebrovascular injury

The low rate of positive imaging findings makes it difficult for physicians to decide whether or not a patient presenting at the emergency department after strangulation requires diagnostic imaging, and if so, what imaging study to request [2, 3, 11]. Some authors maintain that routine computed tomography angiography (CTA) may not be justified in alert patients (i.e. Glasgow Coma Scale score of 15) after strangulation [2, 3, 13]. There is an argument that the high costs of CTA and both the risks related to radiation and to adverse reactions to contrast media should be weighed against the low risk of missed injuries [2, 3, 13]. A key difficulty with this approach is that patients with potentially dangerous injuries may present with the same symptoms as those with insignificant injuries [2, 6]. In addition, documenting — even clinically insignificant — injuries may be valuable for medico-legal reasons. In patients with no external injuries (e.g. after chokehold strangulation), imaging may reveal internal injuries [1, 6, 10, 11, 13].

Research in forensic radiology has demonstrated the utility of non-contrast magnetic resonance imaging (MRI) of the neck after survived strangulation for diagnosing and documenting soft tissue injuries, HLC fractures, and BCVI [1, 10, 12]. MRI is the method of choice for assessing abnormalities of the brain, and the preferred modality in terms of radiation safety. Its main drawbacks are limited availability, high costs, long examination times, and the potential for premature MRI termination (e.g. due to claustrophobia).

This study was conducted in a tertiary hospital with approximately 55,000 patient admissions to the emergency department per year. The emergency department routinely refers patients to the radiology department for diagnostic imaging after assault with strangulation. There is an agreement between the two departments that alert strangulation survivors should be referred to MRI. However, depending on patient suitability for MRI, slot availability, and staffing, patients may also be referred for CT.

### Aim of the study

The aims of the study were (1) to assess the type and frequency of injuries in alert patients after non-self-inflicted strangulation (NSIS), (2) to evaluate the accuracy of original radiology reports (ORR) of CT and MRI studies following NSIS compared to expert read-outs (EXR), and (3) to analyze the use and value of imaging modalities.

## Material and methods

This longitudinal, retrospective study included all alert patients who presented to the emergency department of the University Hospital of Bern between January 2008 and December 2020 after non-self-inflicted strangulation and who underwent cross-sectional imaging.

For each case, the following information was retrieved from the electronic medical archive and the picture archiving and communication system: age, sex, strangulation mechanism (i.e. manual, ligature, or not documented), context of strangulation (intimate partner violence, assault by unknown offender, or not documented), type of imaging study performed (CT or MRI), and the ORR. The ORRs had been created by radiology residents and authorized by board-certified radiologists.

Two board-certified radiologists (one neuroradiologist, subspecialized in head and neck imaging and emergency neuroradiology, and one general radiologist, subspecialized in emergency radiology and forensic imaging) reviewed all imaging studies for the presence of soft tissue hematoma (STH), and HLC fracture, BCVI, and hypoxic brain injury (HBI). Imaging findings were noted in consensus. In cases of disagreement, a third board-certified neuroradiologist acted as arbiter. STHs were considered minor injuries, whereas HLC fractures, BCVIs and focal HBI were classified as potentially dangerous injuries. This retrospective expert read-out (EXR) was used as reference standard against which all ORRs were compared.

### Statistical analysis

Continuous variables, such as patient age, are expressed as means, medians, and ranges. Categorical variables and qualitative parameters such as patient sex, strangulation mechanism, context of strangulation, type of imaging study performed, and number of injuries reported in ORR and EXR are expressed as frequencies and percentages. Distributions of categorical variables were compared with the chi-square goodness-of-fit test and independence of categorical variables was tested with a chi-square test of independence. Sensitivity, specificity, positive predictive value, negative predictive value, and accuracy of the ORRs were calculated. Statistical analysis was performed with SocSciStatistics [14]. A *p* value of < 0.05 was used as the threshold for statistical significance.

## Results

### Study population

A total of 116 cases of NSIS were reported during the 13-year study period (mean: 9 NSIS patients per year, or 1 NSIS patient every 6 weeks). Mean patient age was 33.8 years (range 16–75, median 30). There was significant asymmetry between the sexes (82 females (71%); 34 males (29%), *p *< .001, *χ*^2^).

Manual strangulation was the predominant mechanism (*n* = 113, 97%; ligature *n* = 1, 1%; not documented *n* = 2, 2%). The context of NSIS was intimate partner violence in 53% (*n* = 62; 61 females, 1 male, *p *< .001, *χ*^2^), assault by unknown offender in 35% (*n* = 40; 30 males, 10 females, *p *< 0.002, *χ*^2^), and not documented in 12% of cases (*n* = 14, 11 women, 3 men, *p*=.033, *χ*^2^) (see Table 2 for more details).

**Table 2:** Study population stratified by patient sex

		All		Women		Men	
		116	100%	82	71%	34	29%
Age		33.8 (16–75, med. 30)		33.8 (16–75, med. 30)		33.4 (76–65, med. 30)	
Strangulation	Manual	113	97%	79	96%^a^	34	100%^b^
	Ligature	1	1%	1	1%^a^	0	0%^b^
	n/d	2	2%	2	2%^a^	0	0%^b^
Context	IPV	62	53%	61	74%^a^	1	3%^b^
	ABUO	40	35%	10	12%^a^	30	88%^b^
	n/d	14	12%	11	13%^a^	3	9%^b^

*med.*, median; *IPV*, intimate partner violence; *ABUO*, assault by unknown offender; *n/d*, not documented. ^a^% of females (82 = 100%); ^b^% of males (34 = 100%)

### Imaging modality

A total of 132 imaging studies were performed (CT: *n* = 67, 51%; MRI: *n* = 65, 49%). A few patients underwent both CT and MRI (16/116, 14%). CT angiography of the head and neck (*n* = 40, 60% of CTs) and non-contrast-enhanced MRI of the neck (*n* = 38, 58% of MRIs) were the most frequently ordered studies (see Table 3 for more details).

**Table 3:** Use of imaging modalities and imaging protocols of all 132 studies

Imaging protocol	CT (*n* = 67)		MRI (*n* = 65)	
Angiography head and neck	40	60%^a^	23	35%^b^
Angiography neck	8	12%^a^	3	5%^b^
Head and neck W contrast	6	6%^a^	0	0%^b^
Neck W contrast	2	3%^a^	0	0%^b^
Head and c-spine WO contrast	3	4%^a^	1	2%^b^
Neck WO contrast	0	0%^a^	38	58%^b^
Head WO contrast	6	6%^a^	0	0%^b^
Facial bones WO contrast	2	3%^a^	0	0%^b^

*W contrast*, with contrast; *c-spine*, cervical spine; *WO contrast*, without contrast*. *^a^% of CT studies (67 = 100%); ^b^% of MRI studies (65 = 100%)

### Imaging findings

Most of the 116 patients had no abnormality on imaging after NSIS (no injury: 82/116, 71%; injury visible: 34/116, 29%). Minor injuries (i.e. STH) were observed in 26/116 patients (22%) and 8/116 patients (7%) had potentially dangerous injuries (i.e. HLC fracture, BCVI, or focal HBI). Injuries in two injury categories were recorded in 6/34 patients with injuries; 28/34 patients had injuries in one injury category, resulting in a total of 40 injuries across all imaging studies. Discrepancies between EXR and ORR were found in 21/116 patients (18%) or 21/34 patients with injuries (62%). Of these, 90% (19/21) were false negatives (FN) and 10% (2/21) were false positives (FP). Underreporting of injuries occurred significantly more often in CT than MRI reports (19/27 injuries (70%) not reported following CT versus 5/23 injuries (22%) not reported following MRI, *p *< .001, *χ*^2^).

**Fig. 1:**
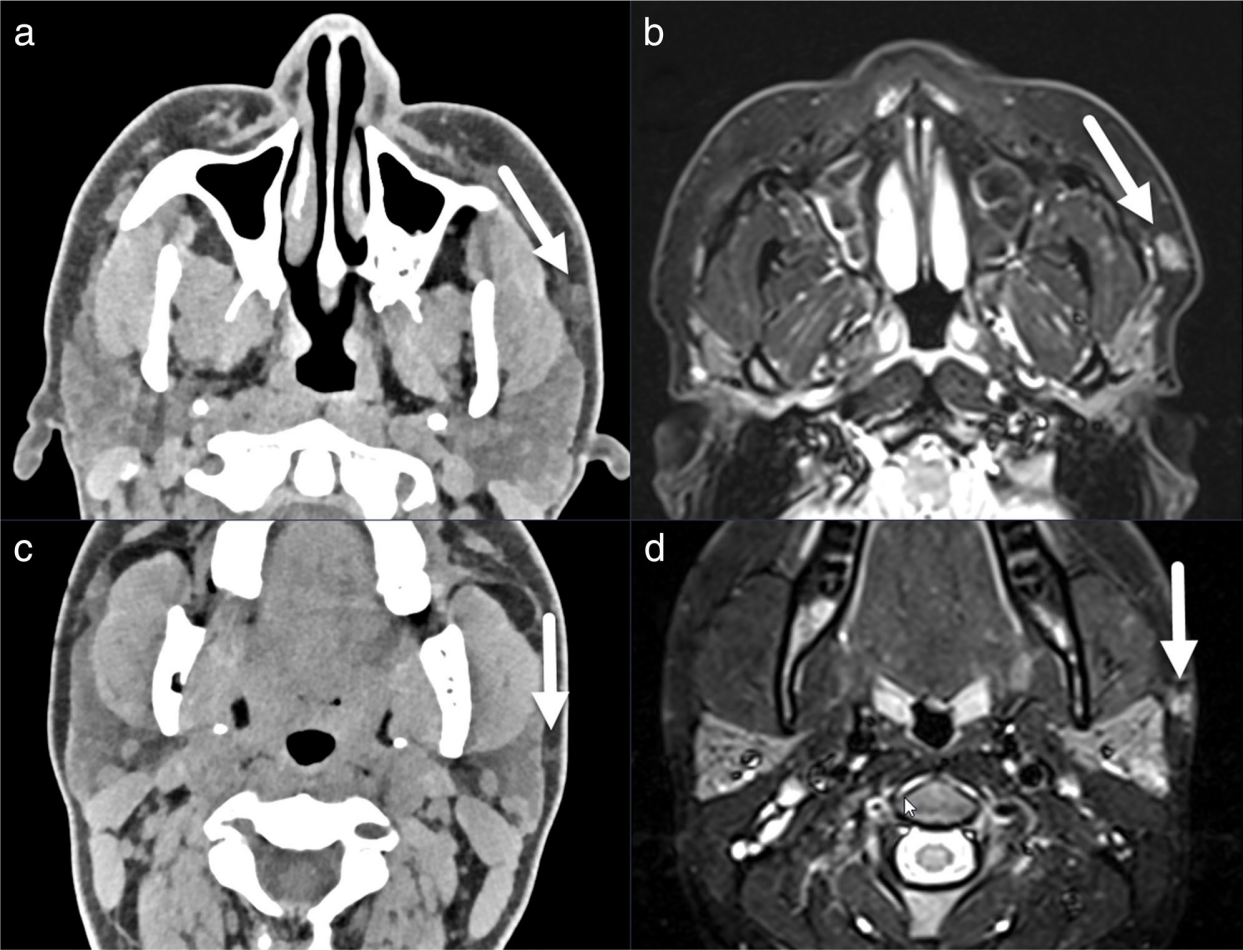
Non-contrast-enhanced axial computed tomography (CT) and magnetic resonance imaging (MRI) turbo inversion recovery magnitude images at the level of the maxillary sinus (**a**) and (**c**) and parotid glands (**b**) and (**d**). Subcutaneous soft tissue hematomas are more difficult to detect on CT (arrows in (**a**) and (**c**)) than on MRI, where even small fluid accumulations provide high signal intensity relative to the surrounding fat (arrows in (**b**) and (**d**))

**Fig. 2:**
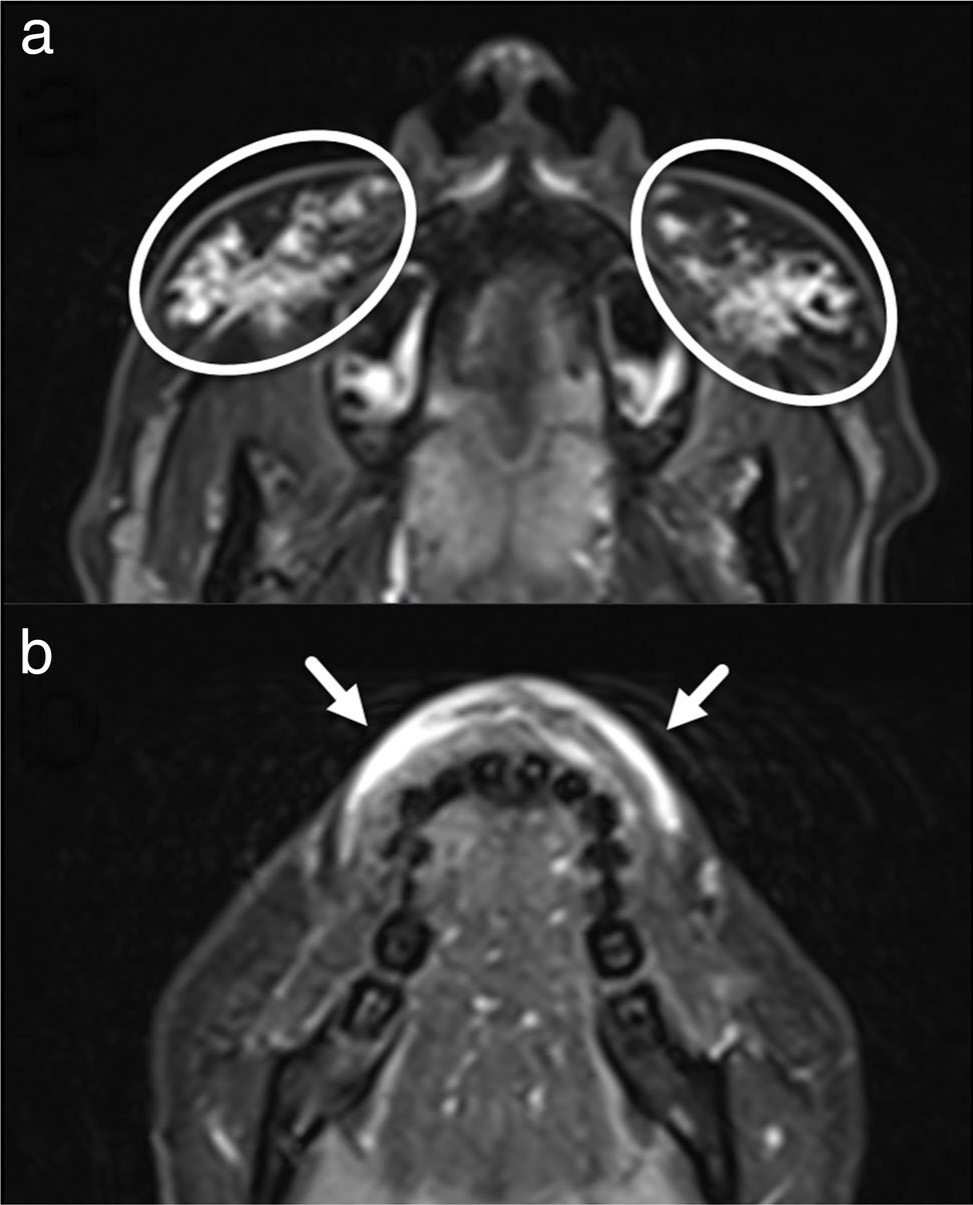
Non-contrast-enhanced axial magnetic resonance imaging (MRI) turbo inversion recovery magnitude images at the level of the maxillary sinuses (**a**) and lips (**b**). Cosmetic dermal filler injections (circles in (**a**) and white arrows in (**b**)) were interpreted as soft tissue hematoma in the original radiology report

**Fig. 3:**
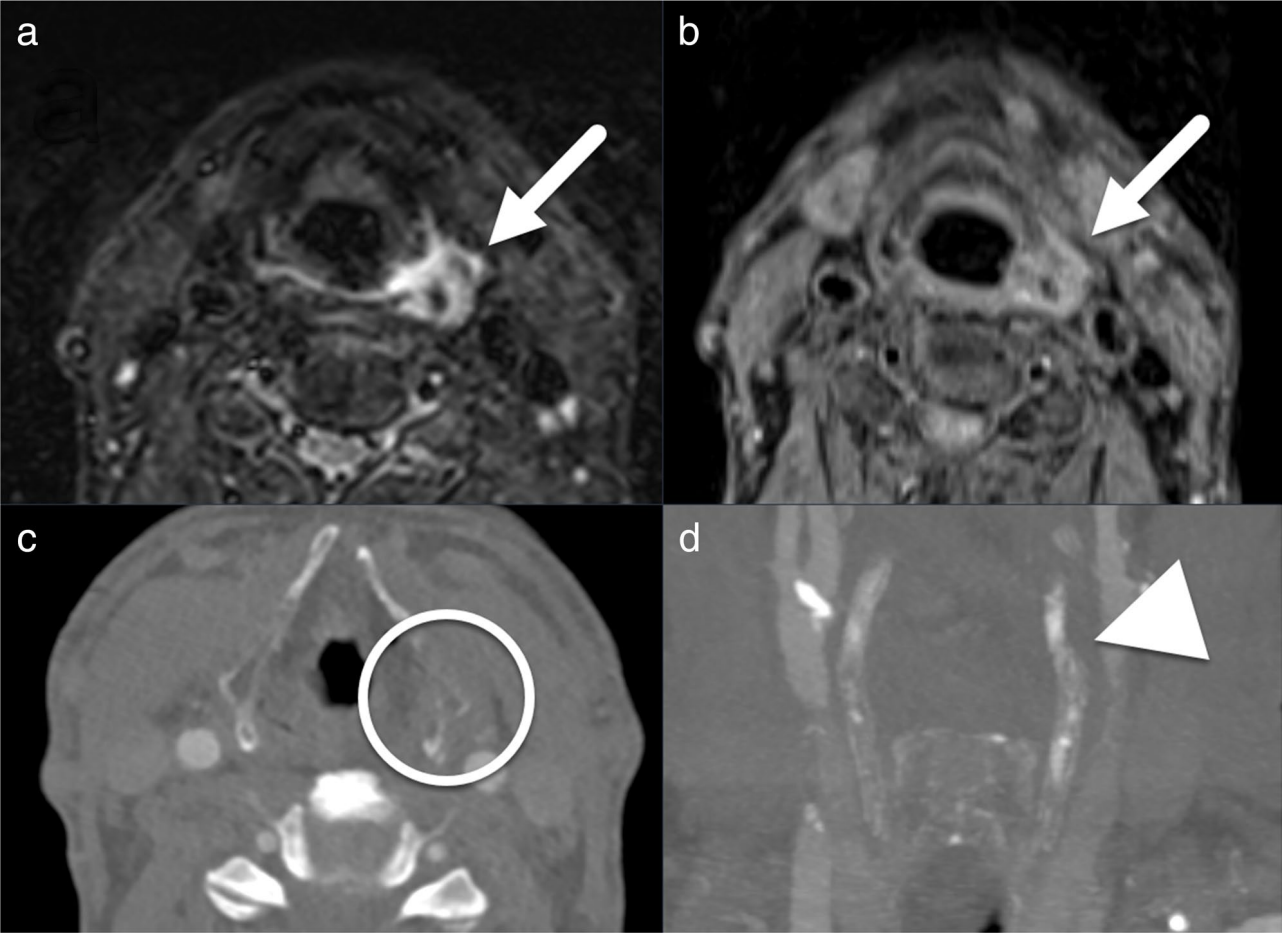
Non-contrast-enhanced axial magnetic resonance imaging (MRI) turbo inversion recovery magnitude (TIRM) (**a**) and T1 fat-saturated (T1fs) (**b**) images and axial computed tomography angiography (CTA) (**c**) and coronal 5-mm maximum intensity projection (MIP) CTA reformat (**d**) at the level of the thyroid cartilage. The focal high signal intensity on TIRM and T1fs images compared to surrounding hypointense fat is useful to identify hematomas caused by fractures of the superior thyroid cartilage horn (white arrows in (**a**) and (**b**)). The level of the fracture is marked with a white circle in (**c**) and an arrowhead in (**d**)

**Fig. 4:**
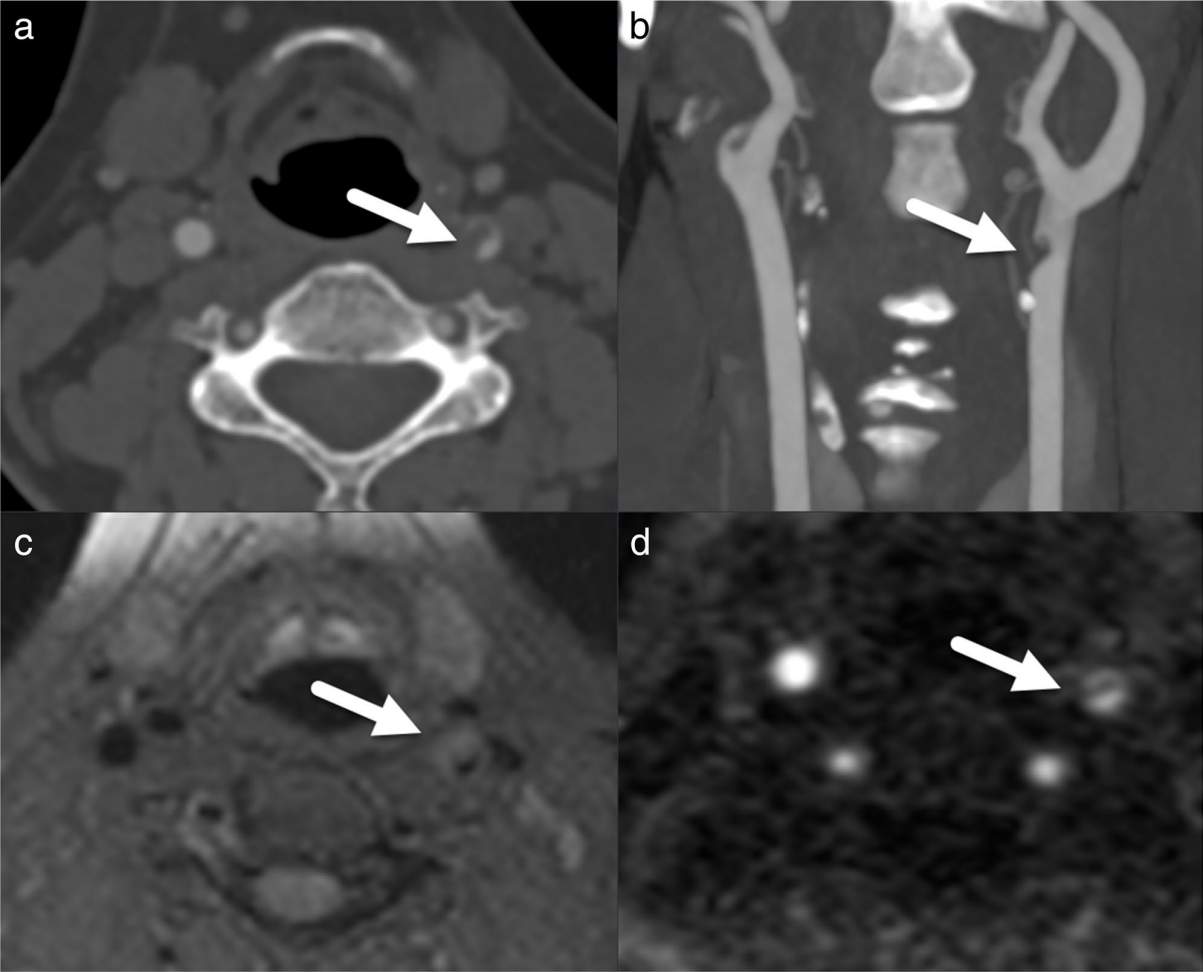
Axial computed tomography angiography (CTA) (**a**) and coronal 5-mm maximum intensity projection (MIP) CTA reformat (**b**) and axial T1 fat-saturated (T1fs) MRI without contrast (**c**) and axial magnetic resonance angiography (MRA) subtraction (**d**) at the level of the dissection of the distal left common carotid artery with intramural hematoma (marked with arrow in all images)

**Fig. 5:**
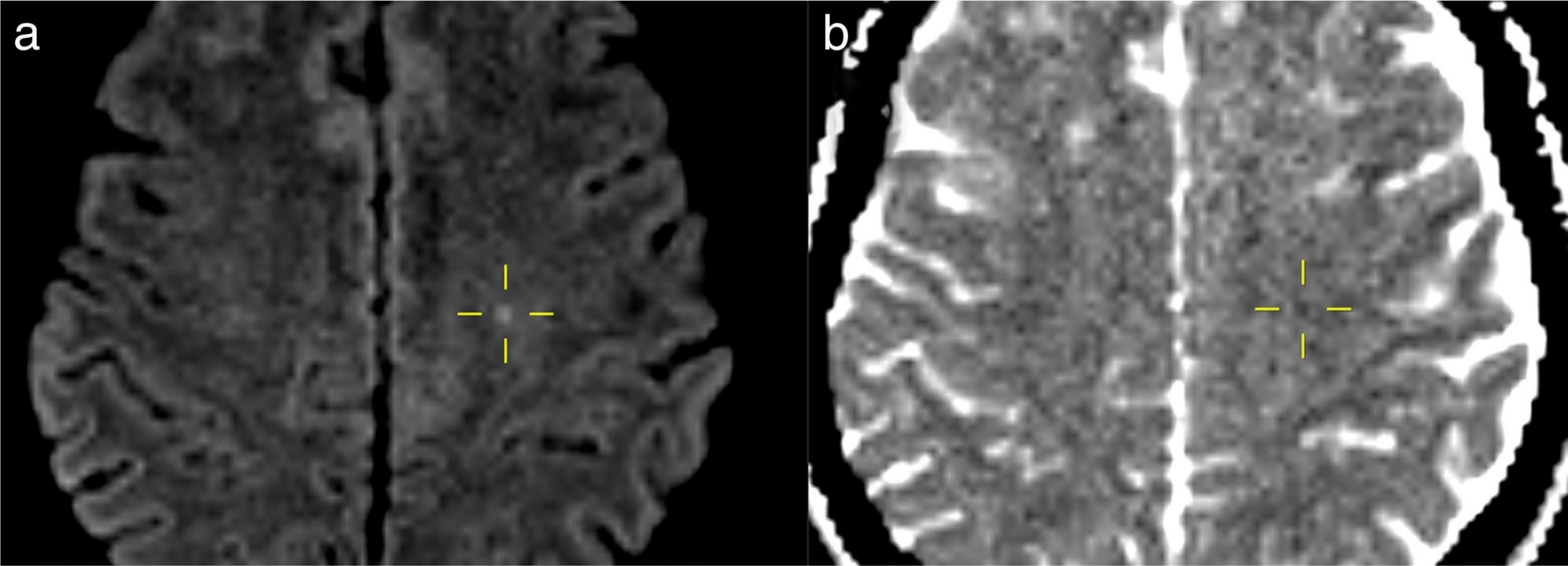
Axial diffusion-weighted image (DWI) (**a**) and apparent diffusion coefficient (ADC) map (**b**). Evidence of punctiform white matter diffusion restriction (marked with crosshairs in both images) consistent with focal hypoxic brain injury (same patient as Fig. 4)

STH was present in 31/116 patients (27%), and discrepancies of ORR occurred in 16 patients (FN 15/31 of cases with STH, 48%; FP 1/85 cases without STH, 1%). Underreporting of STH occurred significantly more often on CT than on MRI (5/20 STH in CT reports (20%) versus 13/16 true positive STH in MRI reports (81%), *p *< .001, *χ*^2^) (Fig. 1). In one case, cosmetic dermal filler injections were misinterpreted as STH on MRI (Fig. 2). HLC fractures were present in 6/115 patients who had imaging of the larynx (5%), of which 3 were missed in the ORR (FN: 3/6, 50%) (Fig. 3). Evidence of BCVI was found in 2/108 patients who had vascular imaging (2%), and discrepancies in ORR occurred in 2 cases (FN 1/2 cases with BCVI, 50%; FP 1/106 cases normal vascular studies, 1%) (Fig. 4). Focal HBI was present in 1/82 patients who had brain imaging (1%) (Fig. 5) (see Table 4 for an overview).

**Table 4:** Number of injuries detected by EXR compared to injuries reported in ORR

	Patients (*n* = 116)			CT reports (*n* = 67)			MRI reports (*n* = 65)		
	EXR	ORR		EXR	ORR		EXR	ORR	
	Present	rep.	not rep.	Present	rep.	not rep.	Present	rep.	not rep.
STH	31	16^a^	15	20	5	15	16	14^a^	3
HLC fracture	6	3	3	6	3	3	5	3	2
BCVI	2	2^a^	1	1	1^a^	1	1	1	0
Focal HBI	1	1	0	0	0	0	1	1	0

*ORR*, original radiology report; *EXR*, expert read-out; *STH*, soft tissue hematoma; *HLC*, hyoid-larynx-complex; *BCVI*, blunt cerebrovascular injury;* HBI*, hypoxic brain injury; *rep.*, reported in ORR; *not* *rep.*, not reported in ORR. ^a^ORR includes 1 false positive finding in this category. Note: 16/116 patients underwent CT and MRI

Injuries in more than one category were present in 6/116 (5%) patients (STH+HLC fracture; *n* = 5: BCVI+focal HBI; *n* = 1).

Sensitivity and specificity of ORR for MRI and CT combined across all findings were 50% and 98%; positive predictive value, negative predictive value, and accuracy were 91%, 80%, and 82%, respectively. Sensitivity and specificity of ORRs for MRI versus CT were 78% and 97% versus 30% and 98%, respectively. Positive predictive value, negative predictive value, and accuracy were 95%, 89%, and 91% for MRI and 89%, 67%, and 70% for CT.

A subanalysis of reported injuries in ORR of the 16 patients who underwent both CT and MRI yielded consistent results: MRI displayed higher sensitivity and specificity than CT (83% and 100% versus 50% and 92%, respectively). Positive predictive value, negative predictive value, and accuracy were 100%, 96%, and 97% for MRI and 100%, 92%, and 92% for CT (see Table 5 for the subset analysis).

**Table 5:** Subanalysis of patients who underwent both CT and MRI

	CT reports (*n* = 16)			MRI reports (*n* = 16)		
	EXR	ORR		EXR	ORR	
	Present	rep.	not rep.	Present	rep.	not rep.
STH	5	3	2	7	7	0
HLC fracture	5	2	3	5	3	2
BCVI	0	0	0	0	0	0
Focal HBI	0	0	0	0	0	0

*ORR*, original radiology report; *EXR*, expert read-out; *STH*, soft tissue hematoma; *HLC*, hyoid-larynx-complex; *BCVI*, blunt cerebrovascular injury; *HBI*, hypoxic brain injury; *rep.*, reported in ORR;* not* *rep.*, not reported in ORR

## Discussion

This analysis of imaging findings after NSIS revealed that CT and MRI were normal in just over 70% of patients but identified potentially dangerous injuries in 7%. The sensitivity of ORRs was nearly 80% for MRI but only 30% for CT. A substantial number of unreported soft tissue hematomas and larynx fractures were identified, particularly on CT.

These results are important because they indicate that MRI is more appropriate than CT for imaging in alert patients after NSIS. They also underline the importance of having radiologists with specialized knowledge in this area to report these cases and improve patient care, as well as informing potential subsequent medico-legal investigations.

The characteristics of the study population and fundamental imaging findings of this study are in line with those of previous studies: Most patients were women (71%) around 30 years of age (mean 33.8). The predominant injury mechanism was manual strangulation (97%) [1–3, 5, 10]. The predominant context of strangulation was intimate partner violence for women (75%) and assault for men (88%) [4], and most strangulation survivors had no (71%) or only minor injuries (21%) [1–3, 5, 10].

Several points are worth discussing in greater depth: This study found substantial underreporting of STH in ORR, especially in CT reports, where 75% of STH were not reported. The lower soft tissue contrast of CT compared to MRI provides a plausible explanation for this finding, but, even on MRI, nearly 20% of STH were not reported. The reason for underreporting minor — medically insignificant — injuries, such as STH, may be that clinical radiologists focus on reporting medically significant injuries, like the cerebrovascular trauma [2, 3]. This aligns with the findings of Heimer et al, who discovered that clinical radiologists detected fewer abnormalities on MRI of NSIS patients than forensic radiologists did. Forensic radiologists are trained to include medically insignificant injuries in their reports, as they may be significant in a medico-legal context [1, 10, 12].

The prevalence of potentially dangerous injuries was 7% in this study (8/116 patients), higher than in most previous studies [1–6, 10, 12] (see also Table 1). Notably, HLC fractures (6 cases; 5% of cases that had imaging of the neck) were rarely reported in previous studies. Only Heimer and Bergin reported larnyx fractures (1/114 and 1/210 cases, respectively), whereas Christe et al, Zuberi et al, and Matusz et al reported no HLC fractures in their study populations [1–3, 6, 10]. The true prevalence of HLC fractures after survived strangulation remains unknown, but the fact that half of all HLC fractures were missed in the ORRs in this study and the low prevalence of reported HLC fractures in previous studies is in keeping with the assumption that HLC fractures are widely underreported on imaging studies [15].

HLC fractures present a significant diagnostic challenge for radiologists as they are rare, problematic to detect on CT of patients with an incompletely or non-ossified larynx, and difficult to differentiate from both old fractures and anatomical variants of the HLC (present in about 70% of individuals) [15–18]. The finding that all 6 HLC fractures in this study were fractures of the thyroid cartilage is in line with the literature on both non-fatal and fatal neck trauma: the thyroid cartilage is the most common site of HLC fractures, followed by fractures of the hyoid bone [15, 17].

During the EXR, experts found it easier to diagnose acute HLC fractures on MRI than on CT. The focal high signal intensity on turbo inversion recovery magnitude (TIRM) and T1-weighted fat-saturated (T1fs) images was useful for identifying the hematoma caused by the fracture (Fig. 3). This observation may be useful to help general radiologists to recognize HLC fractures on MRI.

The prevalence of BCVI and focal HBI was similar to that reported in previous research [1–3, 5, 6]. There were two cases of BCVI (2% of cases that had vascular imaging), of which one patient experienced transient focal HBI (1% of cases that had brain imaging). Both patients fully recovered. The study also found that one BCVI was missed on CTA and an image artefact on CTA was falsely identified as a BCVI in the ORR. The reasons for these discrepancies cannot be determined retrospectively, but the error rates for BCVI are consistent with those in previous studies on non-fatal strangulation [2, 3].

MRI and CT were nearly equally used in this population (65 MRI versus 67 CT). Overall, complete reporting of injuries was significantly higher with MRI than with CT and the expert readers preferred MRI to CT for diagnosing HLC fractures. Although contrast-enhanced CTA or MRA represent the standard imaging protocols for BCVI in many institutions, there is a high level of evidence regarding the diagnostic value of non-contrast-enhanced MRI for cerebrovascular dissection [19–21] (Fig. 4). The diagnostic accuracy of non-contrast MRI in detecting BCVI is similar to that of contrast-enhanced MRA, particularly for larger vessels like the extra-cranial carotid arteries. However, it is dependent on the specific MRI sequences used for non-contrast vessel visualization and generally demonstrates lower accuracy than contrast-enhanced MRI for smaller arteries [19–21]. 3D time-of-flight MRA and 3D black-blood fat-saturated T1 are the preferred sequences for this purpose because of their short acquisition time, large coverage area, strong fat saturation, and excellent delineation of intramural hematomas during the acute and subacute dissection phase [22]. Additional reasons supporting the use of non-contrast MRI after NSIS are lower costs (no contrast medium, no injection kit, no renal function testing) and shorter examination time than for contrast-enhanced MRI [21].

This study has several limitations, including a small study sample and a retrospective design. With only nine radiology referrals per year, imaging studies after NSIS account for <0.05% of all CT and MRI requests from the emergency department (approximately 18,250 per year). Despite previous studies reporting slightly higher numbers [2, 3, 6, 23], imaging referrals after non-self-inflicted strangulation remain infrequent, with an average of <20 cases per year per institution [1–6]. Considering this, the authors of this article argue that the benefits of using MRI to diagnose or exclude potentially dangerous injuries and to document soft tissue hematomas for medico-legal purposes outweigh the associated healthcare expenses in this small and vulnerable population of patients seeking medical care after a strangulation assault.

The low number of NSIS cases restricts the generalizability of the results of this study, but also highlights why most radiologists are unfamiliar with imaging findings after strangulation. This lack of familiarity contributes to the substantial rate of underreporting in ORR. To address this issue, all patients scanned at the authors’ hospital following strangulation are now being double-reported by subspecialized radiologists. The retrospective study design has some additional limitations. A prospective study would be preferable but would be logistically challenging due to the small number of referrals after NSIS and might also introduce research bias among reporting radiologists. A further limitation of the study is its dependence on the electronic medical archive. The authors acknowledge that there were substantial differences in the documentation of strangulation context, mechanism, patient symptoms, and clinical findings. This heterogeneity of clinical information available in the electronic medical archive was one of the reasons why this study focussed on radiology reports and imaging data.

## Conclusions

Radiology referrals for non-self-inflicted strangulation are infrequent, yet cross-sectional imaging studies can reveal potentially dangerous injuries such as larynx fractures, blunt cerebrovascular injuries, or focal hypoxic brain injuries in one of 14 patients.

The substantial number of unreported injuries — especially in patients who underwent CT — emphasizes the need for radiologists with specialized knowledge to report these cases.

Considering the higher accuracy of MRI and the absence of ionizing radiation, MRI appears to be more appropriate than CT for the investigation of injuries after strangulation in alert patients.

## Abbreviations

ADC: Apparent diffusion coefficient
BCVI: Blunt cerebral vascular injuries
CTA: Computed tomography angiography
DWI: Diffusion-weighted imaging
EXR: Expert read-outs
FN: False negatives
FP: False positives
HBI: Hypoxic brain injury
HLC: Hyoid-larynx complex
MRI: Magnetic resonance imaging
NSIS: Non-self-inflicted strangulation
ORR: Original radiology reports
STH: Soft tissue hematoma
TIRM: Turbo inversion recovery magnitude

## Declarations

### Guarantor

The scientific guarantor of this publication is Franca Wagner.

### Statistics and biometry

One of the authors (JH) has significant statistical expertise. However, no complex statistical methods were necessary for this paper.

### Study subjects or cohorts overlap

Some study subjects have been previously reported in: Hostettler-Blunier S, Raoussi A, Johann S, Ricklin M, Klukowska-Rötzler J, Utiger S, Exadaktylos A, Brodmann Maeder M. Domestic Violence at the University Emergency Department Bern: A Retrospective Analysis from 2006 to 2016. *Praxis* 2018; 107: 886–892 https://doi.org/10.1024/1661-8157/a003044.

### Methodology

• retrospective

• observational study

• performed at one institution

## References

[1] Heimer J, Tappero C, Gascho D (2019). Value of 3T craniocervical magnetic resonance imaging following nonfatal strangulation. Eur Radiol.

[2] Zuberi OS, Dixon T, Richardson A, Gandhe A, Hadi M, Joshi J (2019). CT angiograms of the neck in strangulation victims: incidence of positive findings at a level one trauma center over a 7-year period. Emerg Radiol.

[3] Matusz EC, Schaffer JT, Bachmeier BA (2020). Evaluation of nonfatal strangulation in alert adults. Ann Emerg Med.

[4] Williamson F, Collins S, Dehn A, Doig S (2022). Vascular injury is an infrequent finding following non-fatal strangulation in two Australian trauma centres. Emerg Med Australas.

[5] MacDonald Z, Eagles D, Yadav K, Muldoon K, Sampsel K (2021). Surviving strangulation: evaluation of non-fatal strangulation in patients presenting to a tertiary care sexual assault and partner abuse care program. CJEM.

[6] Bergin A, Blumenfeld E, Anderson JC, Campbell JC, Patch M (2022). Describing nonfatal intimate partner strangulation presentation and evaluation in a community-based hospital: partnerships between the emergency department and in-house advocates. J Head Trauma Rehabil.

[7] Saleem GT, Champagne M, Haider MN (2022). Prevalence and risk factors for intimate partner physical violence-related acquired brain injury among visitors to justice center in New York. J Head Trauma Rehabil.

[8] Alessandrino F, Keraliya A, Lebovic J (2020). Intimate partner violence: a primer for radiologists to make the “invisible” visible. Radiographics.

[9] Matoori S, Khurana B, Balcom MC (2020). Intimate partner violence crisis in the COVID-19 pandemic: how can radiologists make a difference?. Eur Radiol.

[10] Christe A, Thoeny H, Ross S (2009). Life-threatening versus non-life-threatening manual strangulation: are there appropriate criteria for MR imaging of the neck?. Eur Radiol.

[11] De Boos J (2019). Review article: Non-fatal strangulation: hidden injuries, hidden risks. Emerg Med Australas.

[12] Yen K, Vock P, Christe A (2007). Clinical forensic radiology in strangulation victims: forensic expertise based on magnetic resonance imaging (MRI) findings. Int J Legal Med.

[13] Lindberg DM (2019) Low rates of injury in neurologically normal strangulation victims,

[14] Statistics Calculators. Available via https://www.socscistatistics.com/tests/. Accessed 28 Aug 2023

[15] Shi J, Uyeda JW, Duran-Mendicuti A, Potter CA, Nunez DB (2019). Multidetector CT of laryngeal injuries: principles of injury recognition. Radiographics.

[16] Becker M, Burkhardt K, Dulguerov P, Allal A (2008). Imaging of the larynx and hypopharynx. Eur J Radiol.

[17] de Bakker HM, Warmbrunn MV, van den Biggelaar P, Soerdjbalie-Maikoe V, de Bakker BS (2021). Correction to: Fracture patterns of the hyoid-larynx complex after fatal trauma on the neck: retrospective radiological postmortem analysis of 284 cases. Int J Legal Med.

[18] Ubelaker DH, Cordero QR, Wu Y, Linton NF (2020). Anthropological analysis of trauma in throat bone and cartilage: a review. Forensic Sci Int Synerg.

[19] Cuvinciuc V, Viallon M, Momjian-Mayor I (2013). 3D fat-saturated T1 SPACE sequence for the diagnosis of cervical artery dissection. Neuroradiology.

[20] Cho SJ, Choi BS, Bae YJ, Baik SH, Sunwoo L, Kim JH (2021). Image findings of acute to subacute craniocervical arterial dissection on magnetic resonance vessel wall imaging: a systematic review and proportion meta-analysis. Front Neurol.

[21] Edelman RR, Koktzoglou I (2019). Noncontrast MR angiography: an update. J Magn Reson Imaging.

[22] Mehdi E, Aralasmak A, Toprak H (2018). Craniocervical dissections: radiologic findings, pitfalls, mimicking diseases: a pictorial review. Curr Med Imaging Rev.

[23] Bordia R, Freeman C, Kou HH, Culhane J (2022). Do we need neuroimaging in every case of near-hanging?: Experience from a level 1 trauma center and analysis of the National Trauma Data Bank. Emerg Radiol.

